# Low documentation of chronic kidney disease among high-risk patients in a managed care population: a retrospective cohort study

**DOI:** 10.1186/1471-2369-10-25

**Published:** 2009-09-16

**Authors:** Idris Guessous, William McClellan, Suma Vupputuri, Haimanot Wasse

**Affiliations:** 1 Department of Epidemiology, Rollins School of Public Health, Emory University, Atlanta, Georgia, USA; 2 Institute of Social and Preventive Medicine (IUMSP), University of Lausanne, Lausanne, Switzerland; 3 Emory University School of Medicine, Renal Division, Atlanta, Georgia, USA; 4 The Center for Health Research, Kaiser Permanente Georgia, Atlanta, Georgia, USA

## Abstract

**Background:**

Early detection of chronic kidney disease (CKD) is sub-optimal among the general population and among high risk patients. The prevalence and impact of major CKD risk factors, diabetes (DM) and hypertension (HTN), on CKD documentation among managed care populations have not been previously reported. We examined this issue in a Kaiser Permanente Georgia (KPG) CKD cohort.

**Methods:**

KPG enrollees were included in the CKD cohort if they had eGFRs between 60 and 365 days apart that were <90 ml/min during 1999-2006. The current analysis is restricted to participants with eGFR 10-59 ml/min/1.73 m^2^. CKD documentation was defined as a presenting diagnosis of CKD by a primary care physician or nephrologist using ICD-9 event codes. The association between CKD documentation and DM and HTN were assessed with multivariate logistic regression models.

**Results:**

Of the 50,438 subjects within the overall KPG CKD cohort, 20% (N = 10,266) were eligible for inclusion in the current analysis. Overall, CKD diagnosis documentation was low; only 14.4% of subjects had an event-based CKD diagnosis at baseline. Gender and types 2 diabetes interacted on CKD documentation. The prevalence of CKD documentation increased with the presence of hypertension and/or type 2 diabetes, but type 2 diabetes had a lower effect on CKD documentation. In multivariate analysis, significant predictors of CKD documentation were eGFR, hypertension, type 2 diabetes, congestive heart failure, peripheral artery disease, statin use, age and gender. CKD documentation was lower among women than similarly affected men.

**Conclusion:**

Among patients with an eGFR 10-59, documentation of CKD diagnosis by primary and subspecialty providers is low within a managed care patient cohort. Gender disparities in CKD documentation observed in the general population were also present among KPG CKD enrollees.

## Background

The prevalence of chronic kidney disease (CKD) is increasing in the United States [1,2] and is associated with an increased risk of cardiovascular events, end-stage renal disease (ESRD) and mortality [3-5]. Early identification and appropriate care of CKD patients may delays progression to ESRD and decreases mortality, morbidities, and cost [6,7]. Yet, the level of CKD awareness is low in both the general U.S. population (24%) and among high risk patients (less than 10% among individuals with Stage 3 CKD) [8,9].

The National Kidney Foundation's Kidney Disease Outcome Quality Initiative (KDOQI) evidence-based clinical practice guidelines for CKD recommend periodic screening of high risk individuals for CKD [10]. Specifically, this entails a clinical evaluation (eg. assessment of symptoms, risk factors, medical history, family history), measurement of blood pressure, and laboratory evaluation (eg. serum creatinine, protein-to-creatinine ratio, imaging of the kidneys).

Despite these recommendations, there is evidence from national population surveys that high risk patients are often not screened for CKD and that the presence of CKD may go unidentified [8,11]. Moreover, CKD identification by health care providers within health maintenance organizations have not been fully examined, and predictors of provider awareness of CKD in a high risk patient population have not been reported.

While CKD documentation may not wholly reflect the provider's understanding of the individual patient's clinical status, incorporation of this information into clinical decision systems supported by claims data is important. This can be illustrated by the recent Centers for Medicare and Medicaid Services (CMS) contract awarded to Medicare Quality Improvement Organizations (QIO) in ten states in August of 2008 to reduce disparities in the early detection and care of diabetic CKD among Medicare beneficiaries [12]. A fundamental feature of the chronic disease model is using ongoing surveillance information, such as claims data, to develop CKD quality of care reports for providers and identify opportunities to improve care [13]. If these claims-based data are systematically biased or inaccurate due to incomplete documentation then one can anticipate marked inefficiencies in this and similar efforts to reduce the burden of CKD.

The aims of the present study were to explore these issues by examining the prevalence of a documented CKD diagnosis in high risk diabetic and hypertensive patients by primary and subspecialty providers in a health maintenance organization and to determine the nature and magnitude of predictors of CKD documentation.

## Methods

### Cohort determination

Kaiser Permanente Georgia is a managed care organization that serves racially (40% African Americans) and ethnically diverse patients in the state of Georgia and provides health care for approximately 275,000 people in 2007. Kaiser Permanente Georgia uses electronic medical records for collecting pharmacy and laboratory information, hospitalization records, and outpatient diagnoses. Each member is assigned a unique identification number and all encounters and claims related to a given enrollee include that unique identification number and the date of service.

The KPG Chronic Kidney Disease cohort includes members of Kaiser Permanente Georgia who were identified between January 1, 1999 through January 1, 2006 with an estimated glomerular filtration rate (eGFR) measure <90 ml/min/1.73 m^2^. Patients who had at least 2 estimated glomerular filtration rate (eGFR) measures <90 ml/min/1.73 m^2^, when measured at least 60 and no more than 365 days apart, were included in this study. The date of the first GFR measure was used as the baseline date of entry into the cohort. The Institutional Review Board of Kaiser Permanente Georgia and Emory University approved this study.

### Assessment of eGFR, CKD, and other covariates

The KPG Chronic Kidney Disease dataset includes information on age, gender, health care providers (such as primary care physician or specialist), medications and laboratory measures (such as eGFR). A member was defined as having diabetes mellitus if (s)he had an HbA1c measurement ≥ 7.0% or any of the following criteria within a 12 month period: 1) 2 or more primary care visits with an ICD-9 diagnosis code of 250.xx; 2) 2 or more dispensings of an oral hypoglycemic, insulin, or blood glucose testing strips; or 3) 1 primary care visit with an ICD-9 diagnosis code of 250.xx and 1 dispensing of an oral hypoglycemic, insulin, or blood glucose testing strips. A member was defined as having hypertension if (s)he had blood pressure measured at ≥ 140 SBP or ≥ 90 DBP on a primary care visit or any of the following criteria within a 12 month period: 1) 2 or more primary care visits with an ICD-9 diagnosis code of 401.xx; or 2) 1 primary care visit with an ICD-9 diagnosis code of 401.xx and at least 1 dispensing of a diuretic, ACE inhibitors (ACEI) or angiotensin receptor blockers, beta-blocker, or calcium channel blocker in the previous 12-month period. Health care providers included primary care departments (family medicine, internal medicine, obstetrics/gynecology, urgent care/after hours) and sub-specialty departments (cardiology, endocrinology, nephrology, renal dialysis, hospitalizations). Medication claims data were obtained from the pharmacy dataset that included National Drug Codes, prescription date, quantity, days supply, and fill number. Drugs were then coded and grouped appropriately using the standardized Unified Medical language System (UMLS) [14]. Laboratory test claims required identifying and pulling specific laboratory test codes, the accompanying laboratory test results, as well as the test date. Notably, creatinine tests and results were reported uniformly and eGFR computed. GFR was estimated using the four variables formula from the modification of diet in renal disease study (MDRD) [15].

Because race was missing on 65% of the members, we used a single imputation method based on census-based measures of race. Residential street addresses were geocoded to the census block data from the 2000 U.S. Census. A variable for neighborhood-level percent African-American race was created. The race term in the MDRD formula was then calibrated for percent African-American race. For example, the 1.1212 factor for African Americans in the MDRD formula was adjusted according to the proportion of African Americans (pAA) in the Census block using the formula: 1+(0.212*pAA).

CKD diagnosis documentation was defined by an in-patient or out-patient diagnosis of CKD by primary care or subspecialty physician (both internal Kaiser physicians and external contracted physicians were included). CKD was defined using *International Classification of Diseases, 9^th ^Revision, Clinical Modification *(ICD-9-CM) codes from event encounters. The list of ICD-9 codes used is provided in **Additional Table 1 [Additional file **1** - STable 1]**. To minimize misclassification on CKD status, the current analysis was restricted to members with an eGFR 10-59 ml/min/1.73 m^2^. Members with a diagnosis of end-stage kidney disease were also excluded.

### Statistical analysis

Univariate analyses were conducted using Chi-square tests and student unpaired independent *t *tests. Baseline characteristics evaluated include those previously reported to be important variables predicting CKD incidence and outcomes. Age and eGFR were evaluated as continuous variables, whereas all other variables were dichotomized. Estimated GFR was also categorized by 10-point sub-categories (i.e. eGFR 10-19, 20-29, 30-39, 40-49, 50-59 ml/min/1.73 m^2^). Continuous variables are reported as mean ± SD. Odds ratios and 95% confidence intervals were reported for both diabetes status and CKD identification.

Univariate and multivariable logistic regression models were constructed to examine factors associated with CKD identification. Variables significant at the 5% level from the univariate analyses were included in the logistic regression model. Independent variables were retained in multivariate model unless P > 0.05, or if their inclusion resulted in a change of 10% or more in the estimate of the main effect. All test were two-tailed and statistical significance was set at P < 0.05. We assessed the potential effect modification of independent variables on the association of diabetes status and CKD documentation by performing tests of statistical interaction with a significance level set at P < 0.10. All analyses were performed using SAS version 9.1.3 (SAS Institute, Cary, NC, USA).

## Results

### Study population and baseline characteristics

**Figure 1:**
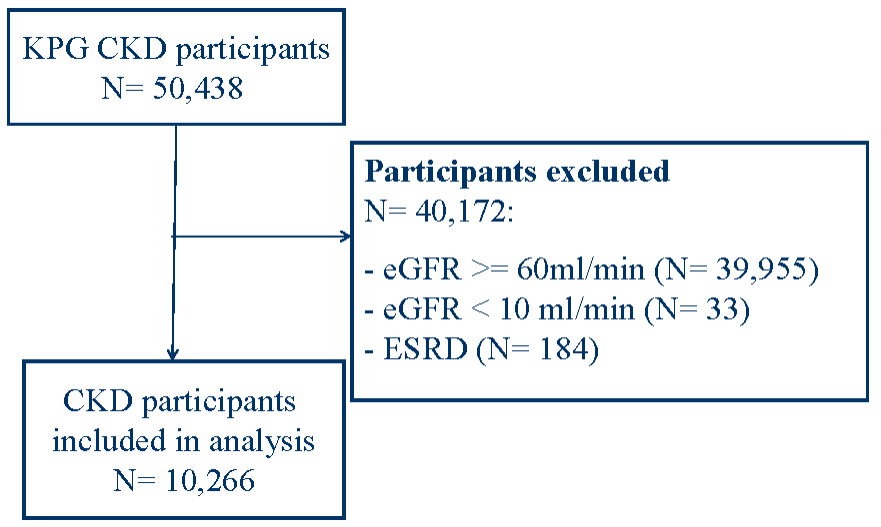
**Study Flow Chart**.

Of the 50,438 members in the KPG Chronic Kidney Disease dataset, 40,172 were excluded because they had an eGFR ≥ 60 (N = 39,955), less than 10 ml/min/1.73 m^2 ^(N = 33), or had a diagnosis of ESRD (n = 184). One fifth of the cohort (N = 10,266) were eligible for the current analysis (Figure 1). Baseline characteristics of the cohort and the distribution by diabetes status are detailed in Table 1.

**Table 1:** Characteristics of 10,266 KP Chronic Kidney Disease Enrollees and Univariate Associations With Type 2 Diabetes Status

Baseline Characteristics	Total Cohort N = 10266 (100) (% or SD)	Diabetic CKD N = 2508 (24.43)(% or SD)	Non diabetic CKD N = 7758 (75.56) (% or SD)	Odds Ratio (diabetic CKD vs non diabetic CKD)	P value
Mean age (continuous)	63.06 (13.43)	63.50 (11.63)	62.92 (13.96)	NA	0.0386
Mean eGFR* (continuous)	48.81 (10.11)	46.04 (11.46)	49.57 (9.52)	NA	< .0001
Men	4155 (40.47)	1111 (26.74)	3044 (73.26)	1.23 (1.12-1.34)	< .0001
Hypertension	7916 (77.11)	2297 (29.02)	5619 (70.98)	4.14 (3.56-4.81)	< .0001
eGFR 50-59	5868 (57.16)	1220 (20.79)	4648 (79.21)	1.00 (Referent)	-
eGFR 40-49	2593 (25.26)	657 (25.34)	1936 (74.66)	1.29 (1.16-1.44)	-
eGFR 30-49	1142 (11.12)	367 (32.14)	775 (67.86)	1.80 (1.57- 2.07)	-
eGFR 20-39	457 (4.45)	171 (37.42)	286 (62.58)	2.27 (1.86- 2.78)	-
eGFR 10-19	206 (2.01)	93 (45.15)	113 (54.85)	3.13 (2.36- 4.15)	< 0.0001**
Coronary artery disease	1335 (13.00)	451 (33.78)	884 (66.22)	1.70 (1.50-1.92)	< .0001
Chronic heart failure	750 (7.31)	293 (39.07)	457(60.93)	2.11 (1.81-2.46)	< .0001
Cerebrovascular disease	163 (1.59)	62 (38.04)	101 (61.96)	1.92 (1.39-2.64)	< .0001
Peripheral artery disease	490 (4.77)	253 (51.63)	237 (48.37)	3.56 (2.96-4.27)	< .0001
ACEI use	1500 14.61)	631 (42.07)	869 (57.93)	2.66 (2.37-2.98)	< .0001
ARB use	156 (1.52)	71 (45.51)	85 (54.49)	2.63 (1.91-3.61)	< .0001
Statin use	1448 (14.10)	476 (32.87)	972 (67.13)	1.63 (1.45-1.84)	< .0001
NSAID use	1213 (11.82)	228 (18.80)	985 (81.20)	0.68 (0.59-0.80)	< .0001

*eGFR in ml/min/1.73 m^2^; ** P value for trend

The mean (SD) age of members was 63 years and mean (SD) eGFR was 48 ml/min/1.73 m^2^. Males constituted 40% of the population and 57% of members had an eGFR between 50 and 59 ml/min/1.73 m^2^. At the time of enrollment hypertension was present for 77%, 24% had diabetes, 13% had coronary artery disease, 7% had congestive heart failure, 5% had peripheral artery disease and 2% had cerebrovascular disease. ACEI and statin medication prescription use were similar (14%). Although all participants had CKD, twelve percent were prescribed a non-steroidal anti-inflammatory medication.

Diabetic subjects were more likely to be older than non-diabetics (63.50 ± 11.63 vs. 62.92 ± 13.96, p = 0.0386), and have a lower mean eGFR than non-diabetics (46.0 vs 49.6 ml/min/1.73 m^2^, p < .0001). The trend for increasing odds of being diabetic with decreasing eGFR was also significant (1.00 (ref) for eGFR 50-59; 1.29 (1.16-1.44) for eGFR 40-49; 1.80 (1.57- 2.07) for eGFR 30-49; 2.27 (1.86- 2.78) for eGFR 20-39, and 3.13 (2.36- 4.15) for eGFR 10-19; *P *for trend < .0001).

Diabetic participants were 4-fold more likely to have hypertension (P < .0001), and were more than 3-fold more likely to have peripheral artery disease (P < .0001). Diabetic subjects were 70% more likely to have coronary artery disease (P < .0001), two-fold more likely to have congestive heart failure (P < .0001)) and 92% more likely to have cerebrovascular disease (P < .0001) compared to non-diabetic patients. Significant differences were also found regarding the use of drug therapies. Diabetic subjects were more likely to receive ACEI, angiotensin receptor blockers, and statin therapy compared to-non diabetic CKD subjects, and 32% less likely to receive NSAIDs (P < .0001).

### Prevalence of CKD documentation

Table 2 describes baseline patient characteristics by CKD documentation. Only 14.4% of subjects, with a baseline GFR between 10- 60 ml/min/1.73 m^2 ^had a CKD diagnosis present at baseline. Subjects with an eGFR < 50 ml/min/1.73 m2, male gender, hypertension, diabetes, coronary artery disease, congestive heart failure, cerebrovascular and peripheral artery disease were significantly more likely to be documented with CKD in the univariate analysis.

**Table 2:** Univariate associations with CKD documentation

Baseline Characteristics	With CKD Documentation N = 1478 (14.40) (% or SD)	Without CKD Documentation N = 8788 (85.6) (% or SD)	Odds Ratio (with vs without CKD documentation)	P value
Mean age (continuous)	62.84 (13.51)	63.10 (13.41)	NA	0.4829
Mean eGFR* (continuous)	38.66 (13.04)	50.52 (8.41)	NA	< .0001
Men	876 (21.08)	3279 (78.92)	2.44 (2.18-2.73)	< .0001
Hypertension	1397 (17.65)	6519 (82.35)	6.00 (4.77-7.54)	< .0001
Diabetes	565 (22.53)	1943 (77.47)	2.18 (1.94-2.44)	< .0001
eGFR 50-59	346 (5.90)	5522 (94.10)	1.00 (Referent)	-
eGFR 40-49	372 (14.35)	2221 (85.65)	2.67 (2.29-3.12)	-
eGFR 30-49	362 (31.70)	780 (68.30)	7.40 (6.27-8.73)	-
eGFR 20-39	252 (50.14)	205 (44.86)	19.61 (15.84-24.3)	-
eGFR 10-19	146 (70.87)	60 (29.13)	38.83 (28.21-53.46)	< 0.0001**
Coronary artery disease	293 (21.95)	1042 (78.05)	1.83 (1.59-2.12)	< .0001
Chronic heart failure	216 (28.80)	534 (71.20)	2.64 (2.23-3.13)	< .0001
Cerebrovascular disease	38 (23.31)	125 (76.69)	1.82 (1.26- 2.64)	0.0011
Peripheral artery disease	151 (30.82)	339 (69.18)	2.83 (2.32-3.46)	< .0001
ACEI use	300 (20.00)	1200 (80.00)	1.61 (1.39-1.85)	< .0001
ARB use	34 (21.79)	122 (78.21)	1.67 (1.13-2.45)	0.0080
Statin use	248 (17.13)	1200 (82.87)	1.27 (1.09- 1.48)	0.0014
NSAID use	122 (10.06)	1091 (89.94)	0.63 (0.52- 0.77)	< .0001

*eGFR in ml/min/1.73 m^2^, ** P value for trend, NA not appropriate

Subjects with CKD documentation were 61% more likely to use an ACEI (P < .0001)), 67% more likely to use an ARB (P = .0080), 27% more likely to use a statin (P = .0014) and 37% less likely to use an NSAID (P < .0001) compared to patients without CKD documentation.

### Multivariate association between predictors and CKD documentation

**Figure 2:**
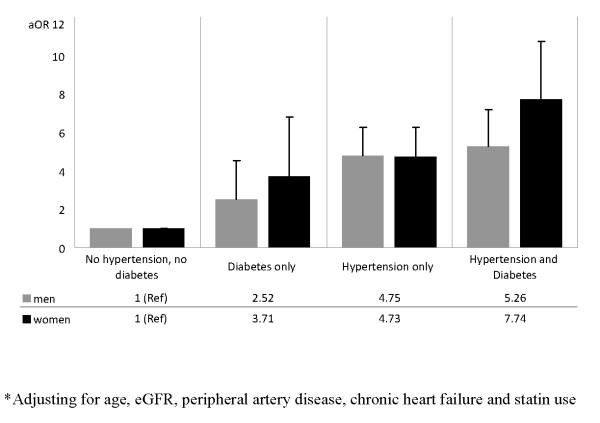
Adjusted Odds Ratio of Chronic Kidney Disease Documentation*.

After controlling for subject characteristics, coronary artery disease and cerebrovascular disease were no longer significant predictors of CKD documentation (Table 3). Older age, lower eGFR, peripheral artery disease, congestive heart failure, and statin use were significantly associated with physician documented CKD. Gender and hypertension were found to be significant effect modifiers of the association between diabetes and CKD documentation (P for interaction = 0.0053 and 0.0065, respectively) therefore adjusted ORs for CKD documentation are presented by gender, hypertension and diabetes status (Figure 2). CKD documentation was more likely among male and female patients with hypertension and/or diabetes. Within each strata, CKD was more likely to be documented among men than women.

**Table 3:** Predictors of Chronic Kidney Disease Documentation in multivariate analysis

CKD Diagnosis Documentation Prediction Variables	aOR of CKD documentation (95%CI)	P value
Age at baseline (by 1 year of increase)	0.98 (0.98-0.99)	< .0001
eGFR** 40-49 vs 50-59	2.55 (2.17-3.00)	< .0001
eGFR 40-39 vs 50-59	7.39 (6.19-8.82)	< .0001
eGFR 20-29 vs 50-59	18.37 (14.59-23.13)	< .0001
eGFR 10-19 vs 50-59	35.33 (25.14-49.65)	< .0001
Coronary artery disease	1.05 (0.87-1.25)	0.5882
Cerebrovascular disease	1.22 (0.80-1.85)	0.3477
Peripheral artery disease	1.51 (1.19-1.93)	0.0008
Chronic heart failure	1.44 (1.18-1.78)	0.0004
Statin use	1.22 (1.03-1.46)	0.0194
ACEI use	0.97 (0.82-1.15)	0.7673
NSAID use	0.81 (0.65-1.01)	0.0705

*Significant interactions in multivariate model were found between type 2 diabetes and gender with respect to CKD documentation (p value for interaction = 0.0053), as well as between type 2 diabetes and hypertension (p value for interaction = 0.0065) with respect to CKD documentation.

**eGFR in ml/min/1.73 m^2^

**Figure 3:**
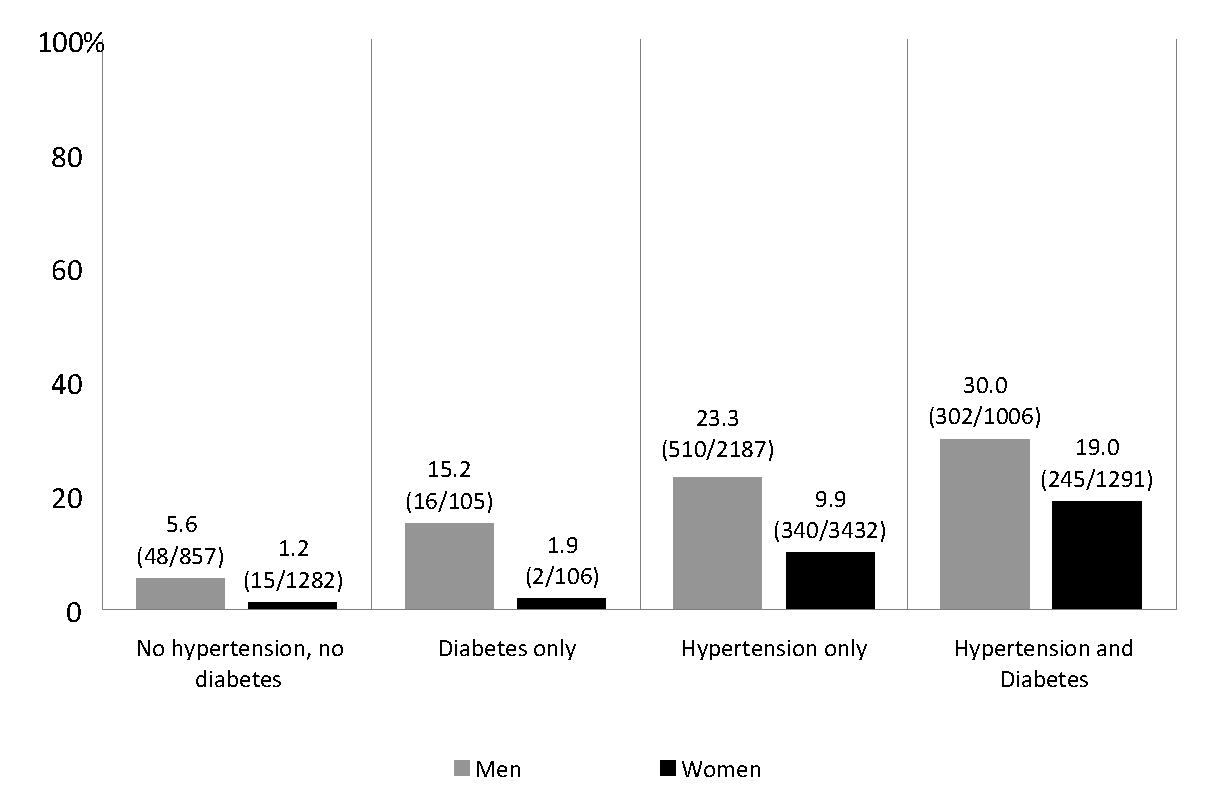
**Prevalence of Chronic Kidney Disease Documentation (%)**.

The prevalence of CKD documentation among CKD participants with hypertension and diabetes were 30% in men and 19% women (Figure 3). Male hypertensive patients were four times more likely to be documented with CKD than non-hypertensive/non-diabetic participants (adjusted OR [aOR], 4.75; 95%CI 3.60 to 6.27)], while those with diabetes were two times more likely to be documented with CKD than non-diabetics (aOR, 2.52; 95%CI 1.40 to 4.53). A similar trend was found among women after adjustment, but the magnitude of the interaction between diabetes and hypertension was greater. Female CKD patients with both diabetes and hypertension were seven times more likely to have a CKD diagnosis documentation than non-hypertensive/non-diabetic participants (aOR, 7.74; 95%CI, 5.58 to 10.72), while males with diabetes and hypertension were five times more likely to have a CKD diagnosis documentation than non-hypertensive, non-diabetic males (aOR, 5.26; 95%CI 3.85 to 7.20).

Sub-analysis of the impact of diabetes status on CKD documentation within each eGFR category was performed. In nearly all strata, the presence of diabetes increased the likelihood of CKD documentation.

## Discussion

Our primary findings are that, among patients with an eGFR 10-59 ml/min/1.73 m^2^, documentation of CKD within a managed care organization is low (14.4%) and that CKD documentation is associated with male gender, increasing age, CKD severity, peripheral artery disease and congestive heart failure. In addition, CKD was more likely to be documented among patients with a combination of hypertension and diabetes.

The prevalence of CKD documented in our analysis is similar to the proportion of patients identified by their primary care physician as having CKD (13.9%) and reported in the pre-intervention period of an educational program performed at an outpatient family medicine practice [16].

Our findings suggest that CKD identification within a managed care organization is as low as in the non-institutionalized U.S. adult population. Indeed, studies assessing self-reported physician diagnosed kidney disease awareness among U.S. adults in the National Health and Nutrition Examination Survey (NHANES) found that CKD identification ranged from 2.9% (women with Stage 3 CKD) to 24.3% (individuals with a GFR of 15 to 59 ml/min per 1.73 m^2 ^and albuminuria) [8,11]. Differences in the estimates reported from the NHANES study and our current study are notable since we assessed physician documentation of CKD using claims diagnoses while the NHANES study assessed self-reported CKD awareness by participants.

We found that CKD documentation by providers increased with the presence of hypertension or diabetes in both genders, but was consistently and considerably lower among women. While CKD was documented in one third of men with hypertension and diabetes, less than 20% of similarly affected women were documented to have CKD. The gender difference in CKD identification was even greater among participants without hypertension or diabetes, hypertension only, and diabetes only. Previous findings suggest that women are less likely to be aware of their CKD status, and our results extend these findings by reporting that providers are less likely to recognize CKD among women with a reduced GFR compared with men [8,11,17]. It has been suggested that health care providers are prone to overlook CKD in women who may have a serum creatinine value in the normal range, due to decreased muscle mass, but have a decline in GFR. Accordingly, the eGFR predictive equation is recommended to estimate the level of renal function from creatinine concentration [18].

We found that eGFR, hypertension, diabetes, congestive heart failure, peripheral artery disease, statin use, and older age were factors significantly associated with CKD diagnosis documentation. CKD patients with hypertension and/or diabetes were more likely to be identified as having CKD than were CKD participants without diabetes or hypertension.

While current guidelines recommend the screening of individuals with diabetes or hypertension for CKD,[6,19] we found that patients with hypertension were more likely be documented as having CKD. Reasons for the greater impact of hypertension on CKD identification are unclear. It is possible that the lack or small number of endocrinologists within the HMO may delay CKD identification in diabetic enrollees, as other providers may not be as aware of the American Diabetes Association Guidelines recommending that serum creatinine is measured annually for eGFR in all adult diabetics [18]. The discrepancy between the effect of hypertension and diabetes has been reported in the general population, where a history of hypertension, but not diabetes, significantly increases the likelihood of CKD awareness by almost 3 times (aOR, 2.98; 95%CI, 1.39 to 6.39) [8].

Our study findings are consistent with previous observations that eGFR is a strong predictor of CKD identification [8,11,20]. We found a significant trend of CKD documentation with decreasing eGFR. Nevertheless, CKD documentation is only 6%, 14%, 31% and 50% among enrollees with eGFR 50-59, 40-49, 30-39 and 20-29 ml/min/1.73 m^2^, respectively. One third of enrollees with an eGFR of 10-19 ml/min/1.73 m^2 ^were not documented as having CKD. This finding suggests CKD identification is poor at both early and advanced stages of CKD.

Although age is a key predictor of CKD, we found that CKD documentation decreased with age [21]. A major obstacle in the identification of CKD with increasing age may be the reliance on serum creatinine measurements as an estimation of eGFR,[22] resulting in an under-identification of CKD among the elderly. Our finding is supported by a recent study of Veterans Administration outpatients 70 years and older, revealing that among CKD Stage 2, 3, and 4 patients, the diagnosis of CKD was documented among only 1.2%, 20%, and 74.6% of patients, respectively, with nephrology consults requested in fewer than 5% of patients [23].

Finally, we found that statin medication use was predictive of CKD documentation, even after adjustment for diabetes, hypertension, and cardiovascular disease. While the predictive nature of statin use has not been previously reported in relationship to CKD care, statin use may be a surrogate marker of better quality of care, and has been proposed as a source of treatment bias in the evaluation of cardiovascular disase outcomes [24,25]. Interestingly, ACEI use was not significantly associated with CKD identification after adjustment. Although ACEI has been shown to reduce the rate of progression of CKD in both diabetics and non-diabetics, [26-28] and although it is identified that ACEI is more effective when instituted early in the course of the CKD, ACEI treatment prevalence in our cohort of CKD HMO enrollees was poor, as described in other settings [29-31]. Furthermore, the prevalence of ACEI use was only slightly higher among enrollees with identified CKD than unidentified CKD. This suggests that factors other than CKD identification contribute to the low ACEI treatment prevalence observed among CKD patients. Contrary to a previous report which showed that CKD diabetic patients are not more likely to be prescribed ACEI than those without diabetes,[30] in our analysis, diabetics with CKD were nearly three-fold more likely to be prescribed ACEI than non-diabetic CKD members, confirming the finding of a previous HMO-base analysis [31].

This study has several limitations. CKD was determined on the basis of ICD 9 codes, leading to the possibility of misclassification. Although previous validation studies suggest that ICD-9 coding for disease identification has sensitivity and specificity greater than 80%,[32,33] others suggest lower performance and methodological issues [34,35]. However, our observations that CKD documentation increased with lower eGFRs, and NSAID prescription use was lower among enrollees with identified CKD, suggest that the use of ICD-CM codes is valid. Second we used ICD9-codes from event encounters which list only the presenting condition for that visit. Thus, in the absence of patient medical record abstraction, the estimate of CKD diagnosis documentation that we report may be an underestimate of the true CKD identification in this population. Third, we lacked data on patient race, and instead, used geocoding for estimating race, which then was used for the eGFR calculation. The validity of geocoding for estimating race has been recently explored. Fremont et al. reported that among 17,500 Medicare enrollees, 92% were successfully identified as either black or other using geocoding [36]. In another study, Glaeser et al also found that geocoding can produce accurate estimates of black race [37]. Experts in health services research suggest that in the absence of direct method, the use of block census geocoding is appropriate [38]. To calculate eGFR in the absence of race, others have assumed that no patients were black or alternatively, have used census data to impute an average constant proportion of black in their study population [9,20]. To test the impact of estimating race using geocoding, we performed all our analysis using eGFR without race and it did not meaningfully change the nature and magnitude of the predictors. Since our cohort is limited to Kaiser Permanent Georgia, our results may not be generalizable to persons in other geographic settings. Although this study has limitations, it examines a large cohort of CKD patients. Finally, because our analyses applied to CKD members identified between January 1, 1999 through January 1, 2006, it is possible that current (2009) documentation of CKD is higher as awareness of CKD in the general population and/or among medical professionals may have increased thanks to advertising and published reports.

## Conclusion

In conclusion, among a large cohort of HMO members, the prevalence of CKD diagnosis documentation is low, and similar to that of the general population. Several important predictors are associated with CKD diagnosis documentation among patients with an eGFR 10-59 ml/min/1.73 m^2^, including age, male gender, severity of renal disease, hypertension, diabetes, congestive heart failure, peripheral artery disease, and statin use.

Emphasis on early detection of kidney disease among HMO members may slow the progression and complications of CKD, therefore strategies aimed to increase CKD early detection are warranted [19,39,40]. In addition, efforts that include focusing on patients in whom CKD identification is poor, such as women, and increasing the use of medications associated with a reduction in CKD progression may limit the number of patients who experience morbidity and mortality related to advanced chronic kidney disease.

## Competing interests

The authors declare that they have no competing interests.

## Authors' contributions

All authors conceived of the study, and participated in its design and coordination and helped to draft the manuscript. IG performed the statistical analysis. IG, HW, WM participated in the interpretation of the statistical analyses. All authors read and approved the final manuscript.

## Pre-publication history

The pre-publication history for this paper can be accessed here:

http://www.biomedcentral.com/1471-2369/10/25/prepub

## Supplementary Material

Additional file 1 **Appendix table **1. Diagnosis Codes used for the Definition of Chronic Kidney Disease Documentation. Click here for file
