# Supplementary material for: Low documentation of chronic kidney disease among high-risk patients in a managed care population: a retrospective cohort study
Source: BMC Nephrol. 2009 Sep 16;10:25. doi: 10.1186/1471-2369-10-25 (PMC2753574; doi:10.1186/1471-2369-10-25)
Supplement: Additional file 1 — Appendix table 1. Diagnosis Codes used for the Definition of Chronic Kidney Disease Documentation. [file 1471-2369-10-25-S1.doc]

**Appendix Table 1** Diagnosis Codes used for the Definition of Chronic Kidney Disease Documentation

| Diagnosis | ICD-9CM codes |
| --- | --- |
| Chronic kidney disease | 585 |
| Proteinuria | 791.0 |
| Unspecified disorder of kidney and ureter | 593.9 |
| Hematuria | 599.7 |
| Malignant hypertensive renal disease with renal failure | 403.01 |
| Benign hypertensive renal disease with renal failure | 403.11 |
| Unspecified hypertensive renal disease with renal failure | 403.91 |
| Malignant hypertensive heart and renal disease with renal failure | 404.02 |
| Malignant hypertensive heart and renal disease with heart failure and renal failure | 404.03 |
| Benign hypertensive heart and renal disease with renal failure | 404.12 |
| Benign hypertensive heart and renal disease with heart failure and renal failure | 404.13 |
| Unspecified hypertensive heart and renal disease with renal failure | 404.92 |
| Unspecified hypertensive heart and renal disease with heart failure and renal failure | 404.93 |
